# Primary carcinoma of the frontal sinus with extensive intracranial invasion: A case report and review of the literature

**DOI:** 10.3892/ol.2014.2032

**Published:** 2014-04-03

**Authors:** HENG-ZHU ZHANG, YU-PING LI, LEI SHE, XIAO-DONG WANG, ZHENG-CUN YAN, NAN ZHANG, EN-XI XU

**Affiliations:** Department of Neurosurgery, Northern Jiangsu People’s Hospital, Clinical Medical College of Yangzhou University, Yangzhou, Jiangsu 225001, P.R. China

**Keywords:** frontal sinus, paranasal sinus, squamous cell carcinoma

## Abstract

Primary carcinoma of the frontal sinus is quite rare, with an incidence of 0.3–1.0% of all paranasal sinus carcinomas. The early diagnosis is often difficult and the condition is often mistakenly considered to involve mucoceles, pyoceles or osteomyelitis. The present study reports the case of a 66-year-old male with squamous cell carcinoma originating in the frontal sinus. The presenting symptoms were a cutaneous nodule on the left side of the forehead and a gradually progressive headache. Magnetic resonance imaging (MRI) demonstrated erosion of the ethmoid sinus, frontal lobe and orbit. The radical resection under frontal craniotomy was performed followed by post-operative radiotherapy. Six months after the surgery, the MRI examinations did not find any recurrence, and the patient currently lives symptom-free. The present study illustrates that frontal sinus cancer should be diagnosed early with caution. Total surgical resection followed by radiotherapy and chemotherapy, used singly or in combination, may result in favorable outcomes. The current study discusses the diagnosis, treatment and prognosis of the present case and reviews the associated literature to emphasize the importance of an early identification of this rare disease.

## Introduction

Primary carcinoma of the frontal sinus, accounting for 0.3–1.0% of all paranasal sinus carcinomas, is extremely rare (1,2). The tumor exhibits a high incidence in older patients (>50 years old), with a male to female ratio of 5:1 (3). The symptoms of paranasal sinus carcinoma are ambiguous and non-specific. This often makes it difficult to diagnose these tumors at the early stage. The histologically predominant type of frontal sinus cancer is squamous cell carcinoma (SCC), which is present in 43–64% of cases (4). The present study reports a rare case of SCC arising in the frontal sinus. A 66-year-old male presented with a subcutaneous nodule and underwent a total resection. A large solid tumor in the frontal sinus was detected following head magnetic resonance imaging (MRI), and this tumor invaded into the dura, frontal lobe, ethmoid sinus and orbit. The relevant literature surrounding this disease is also reviewed in the present study in order to raise the issue of the diagnosis, treatment and prognosis of this disease. The study was approved by the Ethics Committee of the Clinical Medical College of Yangzhou University (Jiangsu, China) and informed consent was obtained from the patient.

## Case report

**Figure 1:**
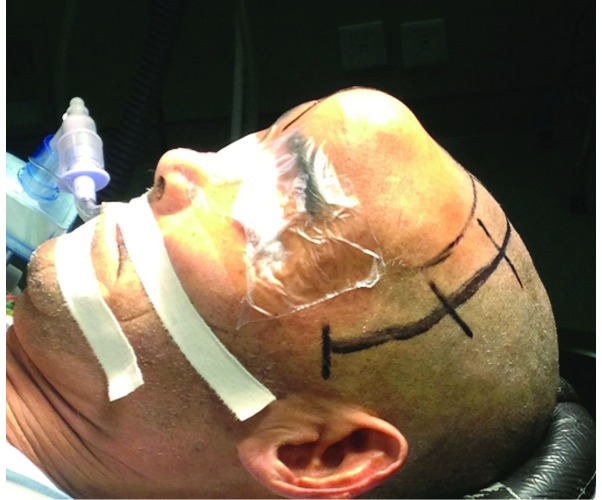
Subcutaneous nodule, 4×6 cm in diameter, on the left side of the forehead..

**Figure 2:**
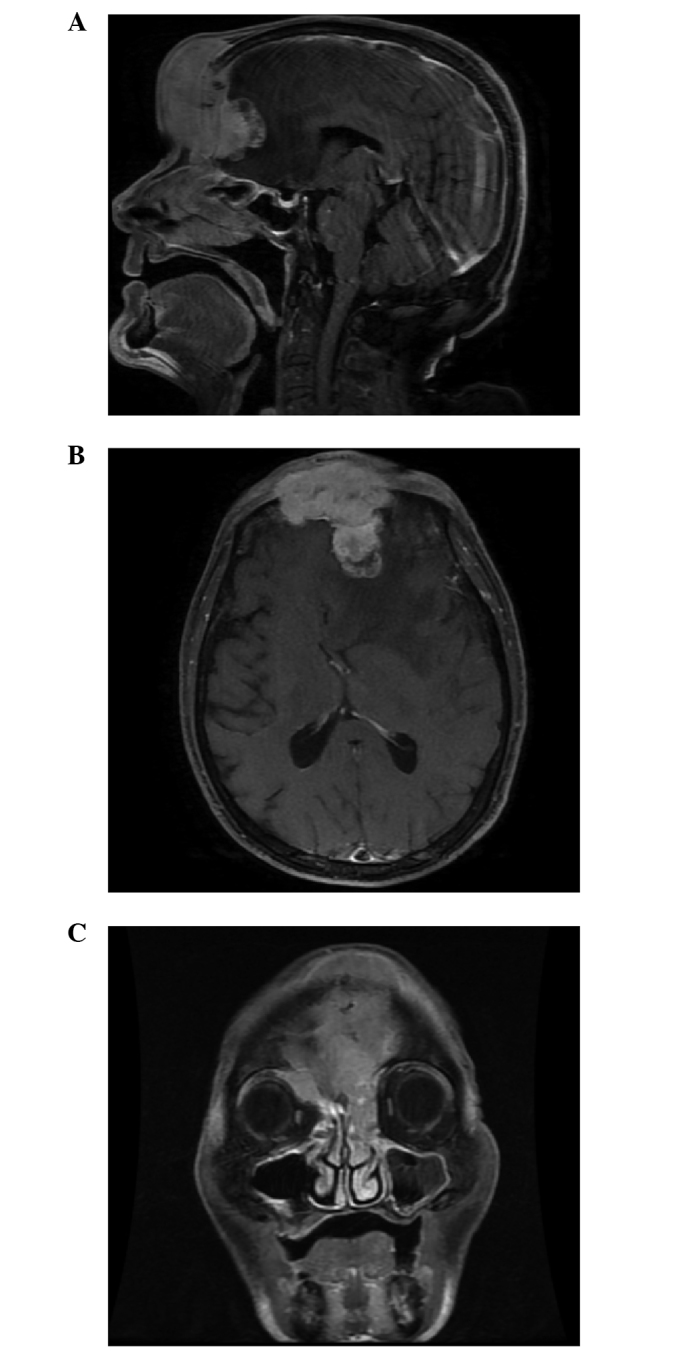
Enhancement of the magnetic resonance imaging (MRI) scan revealing an extensive area of abnormal tissue intensity involving the (A) left frontal sinus, (B) frontal lobe and (C) ethmoid sinus.

A 66-year-old male visited Yizheng People’s Hospital (Yangzhou, China) due to a nodule on the left side of the forehead, which was accompanied by orbital pain and occasional headaches that had been apparent for 4 months. The patient was administered several courses of antibiotics, however, these did not relieve the symptoms. One month prior to the patient being admitted to the Northern Jiangsu People’s Hospital (Yangzhou, Jiangsu, China) the headaches became continuous and extremely severe in the left side of the forehead over the frontal sinus region. The patient was consequently transferred to the Department of Neurosurgery in December 2012. A physical examination revealed a giant, solid and low mobility ~4×6-cm subcutaneous nodule on the left side of the forehead (Fig. 1). The patient could not open the right eye fully due to the nodule. Computed tomography (CT) and MRI of the head showed a large uneven soft-tissue mass measuring 54×76 mm in the left frontal sinus, with intracranial extension into areas that included the frontal lobe, the anterior wall of the ethmoid sinus and the orbit. The signal of the mass was hypointense on T1-weighted MRI images and hyperintense on T2-weighted images. The lesion showed uniform homogenous enhancement following gadolinium-contrast injection (Fig. 2). The patient had no family history of sinus cancer, was not taking any medication, did not smoke and did not drink alcohol. The erosion of the frontal bone gave rise to the suspicion of malignant disease or a metastatic tumor.

**Figure 3:**
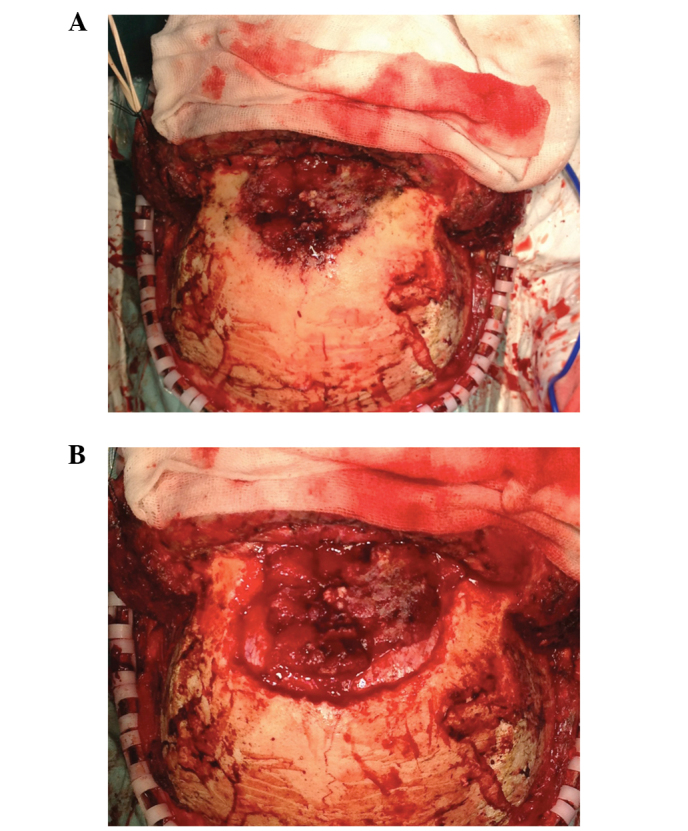
Intraoperative images showing the tumor in the frontal sinus. (A) The tumor was found to erode the frontal bone (4×6 cm). (B) An extended resection into the normal bones and dura was performed to remove a section 7×8 cm in diameter.

A frontal craniotomy was performed with the aim of removing the tumor in December 2012. Subsequent to opening a skin flap, the tumor was found to invade through the frontal bone and to extend to the left frontal lobe (Fig. 3A). The extra-cranial lesion was resected en bloc as widely as possible and the erosion of the frontal bone was extended by resection into the normal bones and dura, to remove a section 7×8 cm in diameter (Fig. 3B). During the surgical procedure, the intra-cranial lesions invading the left frontal lobe, ethmoid sinus, superior sagittal sinus and the wall of the orbit were radically resected. The lesion in the ethmoid sinus was coagulated by electrocauterization subsequent to an extended resection. The frontal base was opened to the ethmoid sinus and sphenoid sinus for drainage to the nasal cavity. The defect of the dura was patched using a Neuro-Patch (DuraMax; Beijing TianXinFu Medical Appliance Co., Ltd., Beijing, China). The forehead and frontal skull base were reconstructed with a rectus abdominis myocutaneous free flap.

**Figure 4:**
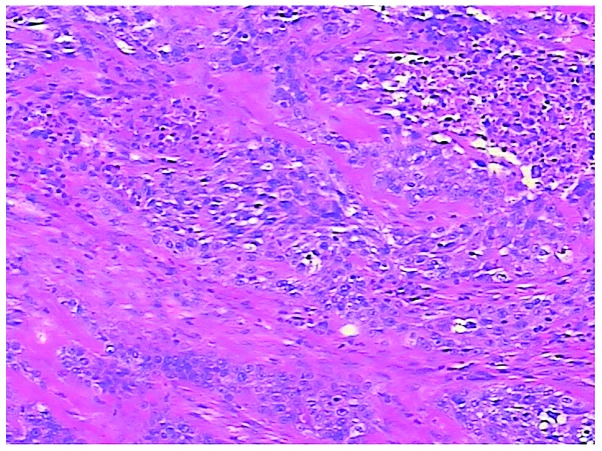
Photomicrograph demonstrating a proliferation of large round cells with abundant cytoplasm and pleomorphic nuclei (HE staining; original magnification, ×100). HE, hematoxylin and eosin.

Histopathological examination of a biopsy revealed a proliferation of large round cells with abundant cytoplasm (Fig. 4). The result of the histopathological examination was a diagnosis of SCC. Given that primary carcinomas of the frontal sinus are rare, the patient underwent further investigations, including chest and abdominal CT scans and a pelvic ultrasound. The results showed that there were no other primary lesions in the other organs. The histological, radiological and investigational results were all diagnostic of a primary carcinoma of the frontal sinus. Four weeks after the surgical procedure, radiotherapy (50 Gy in 15 fractions) was performed. Six months after the surgery, the repeat MRI examinations did not find evidence of recurrence or metastatic lesions.

## Discussion

The incidence of malignant tumors of the anterior skull base appears to be high in the Asian population (5). In 1907, Prawssud (6) was the first to report cell carcinoma of the frontal sinus, and in 1999 Huang (7) was the first to report a case of primary carcinoma of the frontal sinus in China. Paranasal sinus carcinomas present most commonly with nasal obstruction, nasal bleeding, nasal discharge, anosmia, proptosis, diplopia, facial pain, headache and oppressive sensation (8). These symptoms of frontal sinus cancers have been mimicked with other lesions, including mucoceles, pyoceles or osteomyelitis. In the study described by Robinson (4), a unique frontal sinus cancer presented with a form of frontal mucocele. Reddy *et al* (9) also reported a case of frontal sinus cancer mimicking acute frontal sinusitis. In the present case, a patient who originally visited the local hospital’s Department of Dermatology (Yizheng, China) was diagnosed with a benign lesion until the CT scan showed a intracranial invasive lesion. This highlights why these tumors are often only diagnosed at an advanced stage.

An early diagnosis usually depends on the characteristics found upon CT/MRI examination. The pattern of frontal sinus cancers on MRI includes signal hypointensity on T1-weighted images and hyperintensity on T2-weighted images, and always showing homogeneous enhancement. Patients may benefit from the evaluation of the precise extent of frontal sinus tumors, and this may aid in planning the surgical approach and method of skull-base reconstruction. The CT/MRI brain scan may aid in the early detection of frontal sinus SCC, but not confirm it. The pathological results of the resection specimen are required to provide a definite diagnosis. Therefore, we suggest that the criteria of the confirmative diagnosis of frontal sinus cancers is as follows: i) The radiological result shows that the tumor originates from the frontal sinus; ii) the histologically predominant type of tumor sample is SCC, adenocarcinoma or basal cell carcinoma; and iii) there is no positive finding in the investigation of other organs, aiming to eliminate a metastatic tumor. The present case showed a subcutaneous nodule with skin symptoms, including pruritus, pain and numbness. It is important to perform radiological examinations for such patients, aiming to find lesions in the frontal sinus. The diagnosis of a malignant tumor in the frontal sinus should also be considered when patients present with several predictors in the CT/MRI scan, including intracranial invasion, destruction of the anterior skull base and deep orbital spread.

The treatment for frontal sinus tumors remains controversial, and there are no uniform standard treatment strategies. It is consequently difficult for surgeons to choose the correct treatment for frontal sinus tumors due to their rarity and the diversity of treatment. Therefore, conclusions can only be drawn from a limited number of case studies. One previous study revealed that radical resection may be of benefit by significantly alleviating the symptoms and decreasing the recurrence rate (10). A clinical trial reported by Guntinas-Lichius *et al* (11) described the modality and multimodality treatment of 229 patients with malignant sinonasal cancers in a single institution. The study indicated that radical surgery is the treatment of choice for stage I-II tumors. Furthermore, it is also difficult to perform a total excision of frontal sinus cancer due to the complicated anatomy of the frontal sinus, particularly in those patients with wide intracranial invasion. In the present case, the reason for the difficulty in performing a total resection was as follows: i) Prior to the surgery, the tumor had invaded the left frontal lobe, ethmoid sinus, superior sagittal sinus and the wall of the orbit, thereby greatly increasing the difficulty of surgical resection; ii) during the surgery, the border of the nodules was larger than previously observed by the surgeons, resulting in an inadequate resection range; and iii) ethmoid skull base reconstruction was difficult to perform following resection of the lesion. In the present case, radical resection under frontal craniotomy was performed followed by post-operative radiotherapy, and autologous muscle slurry and biological glue were applied to repair the skull base. There was no cerebrospinal fluid leakage and the follow-up MRI imaging showed no recurrence at 6 months.

The adjuvant treatment for this condition is the same as for conventional SCC. It is unclear whether the use of chemotherapy and radiotherapy could improve the prognosis of frontal SCC. The overall response to chemotherapy is ~82%, and regimens based on cisplatin appear to be reasonably effective, particularly in association with 5-fluorouracil and bleomycin (12). A previous study (13) revealed that radiotherapy alone proved ineffective in advanced paranasal sinus carcinomas. By contrast, another study (14) indicated that radiotherapy was helpful in local control. Therefore, analysis of the long-term effects of radiotherapy or chemotherapy by further clinical trials is required to clarify this issue. Currently, the combination of chemotherapy and radiotherapy is considered to be the favored choice. The severe side-effects from radiotherapy, including possible optic nerve damage and radionecrosis, should be taken into consideration. Individual treatment depends on the condition of the patient, therefore, appropriate treatment strategies should be chosen with caution.

With regard to the prognostic situation, previous studies have shown a poor prognosis for this advanced tumor. In 1969, Frew (15) reported 6 cases of frontal sinus cancer, and the average survival duration was only 14 months. Yoshida *et al* (8) reported the case of a 74-year-old male with frontal sinus cancer. Although this patient underwent total resection followed by radiotherapy (50 Gy), the tumor recurred three months after surgery and the patient succumbed 20 months after surgery due to a tumor-associated reason. In China there have only been two case reports of frontal sinus cancer. Huang (7) reported the case of a patient who underwent surgical treatment followed by radiotherapy and chemotherapy. The tumor recurred three months after surgery and the patient succumbed seven months after the surgery due to intracranial metastasis and infection. A study by Wang *et al* (16) reported three cases of frontal sinus SCC; one presented with recurrence along the incision six months after surgery and succumbed 16 months post-operatively, while another tumor recurred in the frontal sinus and orbit 40 days after the surgery and the patient succumbed 10 months after the surgery. The third tumor recurred 14 months after surgery. The study considered that survival with the tumor can be a choice for patients who refuse radical surgical intervention. Despite the performance of radical resection followed by post-operative radiotherapy or chemotherapy, the prognosis of patients with stage III-IV frontal sinus tumors remains extremely poor.

Frontal sinus tumors can easily be mistaken for certain benign lesions, including mucoceles, pyoceles or osteomyelitis. CT/MRI scans are necessary for the early detection of frontal sinus lesions and to aid in the evaluation of the precise extent of the tumors. The present case illustrates the fact that neurosurgeons should try to increase the accuracy of the early diagnosis of suspicious cases using CT/MRI scans, which can aid in the selection of an appropriate therapy. Surgeons should clarify the border of the lesion prior to surgery, and perform the extended resection based on these findings. Therefore, the use of a complete resection with a clear margin and adjuvant therapy is an essential strategy for the treatment of such a malignant tumor.

## Abbreviations

CT: computed tomography
MRI: magnetic resonance imaging
SCC: squamous cell carcinoma
